# Repurposing Approach Identifies Auranofin with Broad Spectrum Antifungal Activity That Targets Mia40-Erv1 Pathway

**DOI:** 10.3389/fcimb.2017.00004

**Published:** 2017-01-18

**Authors:** Shankar Thangamani, Matthew Maland, Haroon Mohammad, Pete E. Pascuzzi, Larisa Avramova, Carla M. Koehler, Tony R. Hazbun, Mohamed N. Seleem

**Affiliations:** ^1^Department of Comparative Pathobiology, College of Veterinary Medicine, Purdue UniversityWest Lafayette, IN, USA; ^2^Department of Chemistry and Biochemistry and the Molecular Biology Institute, University of California, Los AngelesLos Angeles, CA, USA; ^3^Purdue University Libraries, Purdue UniversityWest Lafayette, IN, USA; ^4^Department of Biochemistry, Purdue UniversityWest Lafayette, IN, USA; ^5^Bindley Bioscience Center, Purdue UniversityWest Lafayette, IN, USA; ^6^Department of Medicinal Chemistry and Molecular Pharmacology, College of Pharmacy, Purdue UniversityWest Lafayette, IN, USA; ^7^Purdue Institute for Inflammation, Immunology, and Infectious DiseasesWest Lafayette, IN, USA

**Keywords:** auranofin, antifungal, repurposing, chemogenomic profiling, Mia40-Erv1 pathway

## Abstract

Current antifungal therapies have limited effectiveness in treating invasive fungal infections. Furthermore, the development of new antifungal is currently unable to keep pace with the urgent demand for safe and effective new drugs. Auranofin, an FDA-approved drug for the treatment of rheumatoid arthritis, inhibits growth of a diverse array of clinical isolates of fungi and represents a new antifungal agent with a previously unexploited mechanism of action. In addition to auranofin's potent antifungal activity against planktonic fungi, this drug significantly reduces the metabolic activity of *Candida* cells encased in a biofilm. Unbiased chemogenomic profiling, using heterozygous *S. cerevisiae* deletion strains, combined with growth assays revealed three probable targets for auranofin's antifungal activity—*mia40, acn9*, and *coa4*. Mia40 is of particular interest given its essential role in oxidation of cysteine rich proteins imported into the mitochondria. Biochemical analysis confirmed auranofin targets the Mia40-Erv1 pathway as the drug inhibited Mia40 from interacting with its substrate, Cmc1, in a dose-dependent manner similar to the control, MB-7. Furthermore, yeast mitochondria overexpressing Erv1 were shown to exhibit resistance to auranofin as an increase in Cmc1 import was observed compared to wild-type yeast. Further *in vivo* antifungal activity of auranofin was examined in a *Caenorhabditis elegans* animal model of *Cryptococcus neoformans* infection. Auranofin significantly reduced the fungal load in infected *C. elegans*. Collectively, the present study provides valuable evidence that auranofin has significant promise to be repurposed as a novel antifungal agent and may offer a safe, effective, and quick supplement to current approaches for treating fungal infections.

## Introduction

Invasive fungal infections, particularly those caused by *Candida* and *Cryptococcus*, afflict millions of patients annually resulting in more than 1,350,000 deaths despite the introduction of new antifungal agent (Brown et al., 2012; Pfaller, 2012; Perlin, 2015; Perlin et al., 2015; Sanguinetti et al., 2015). Unfortunately, current antifungal therapies have limited effectiveness in treating invasive fungal infections and suffer from restrictions in route of administration, spectrum of activity, and bioavailability in target tissues such as the brain (Brown et al., 2012; Vandeputte et al., 2012). Further compounding this problem, the development of new antifungal is currently unable to keep pace with the urgent demand for safe and effective new drugs. Hence, there is a pressing and urgent need for novel, inexpensive, and safe antifungal drugs to combat these dangerous pathogens.

The concept of drug repositioning has recently gained momentum and emerged as a viable approach to expedite anti-infective drug development (Butts and Krysan, 2012; Thangamani et al., 2015a,b,c). For example, several reports have demonstrated that auranofin, an orally bioavailable FDA-approved drug for treatment of rheumatoid arthritis, exhibits potent antibacterial and antiparasitic activities (Jackson-Rosario et al., 2009; Debnath et al., 2012; Cassetta et al., 2014; Hokai et al., 2014; Aguinagalde et al., 2015; Thangamani et al., 2016a,b). This discovery led to the FDA granting auranofin Orphan Drug status for treatment of amebiasis. Auranofin is currently approved for long-term treatment of unresponsive rheumatoid arthritis; it is the first, and only, gold compound to be administered orally (Bernhard, 1982; Furst et al., 1983). Although auranofin is slightly less effective than parenteral gold compounds for treatment of rheumatoid arthritis, auranofin's oral bioavailability and reduced associated side effects offer significant advantages over traditional injectable gold drugs (Bernhard, 1982; Furst et al., 1983). Although auranofin has been used clinically for almost 30 years, its mechanism of action (MOA) in treating unresponsive rheumatoid arthritis is still poorly understood (Shaw, 1999; Berners-Price and Filipovska, 2011). The emergence of new anti-rheumatoid drugs with fewer side effects and faster activity has resulted in the decline of oral gold therapy clinically (Berners-Price and Filipovska, 2011). Nevertheless, there has been considerable research efforts employed to identify alternative therapeutic applications for auranofin, particularly in the area of infectious diseases (Shapiro and Masci, 1996; Lobanov et al., 2006; Kuntz et al., 2007; Bonilla et al., 2008; Sannella et al., 2008; Angelucci et al., 2009; Chomont et al., 2009; Jackson-Rosario et al., 2009; Lewis et al., 2011; Caroli et al., 2012; Debnath et al., 2012; Ilari et al., 2012; Chirullo et al., 2013; Tejman-Yarden et al., 2013; Sharlow et al., 2014).

Recent studies by Fuchs et al. (2016) and Stylianou et al. (2014) reported that auranofin also possesses antifungal activity. However, the antifungal MOA and *in vivo* antifungal efficacy of auranofin remain unclear with several possible targets reported. Thus, the objectives of our study were to determine the antifungal activity of auranofin against clinical isolates of different fungal pathogens, to investigate the drug's antibiofilm activity, to deduce auranofin's antifungal MOA using an unbiased chemogenomic approach, and to validate the drug's *in vivo* antifungal efficacy in a *Cryptococcus neoformans*-infected *Caenorhabditis elegans* whole animal model.

## Materials and methods

### Fungal strains and reagents

Fungal strains used in this study are presented in Table 1. Yeast peptone dextrose agar (YPD) was purchased from BD Biosciences (San Jose, CA). Auranofin (Enzo Life Sciences, Farmingdale, NY), fluconazole (Acros Organics, New Jersey), and flucytosine (TCI chemicals, Tokyo, Japan) were purchased from commercial vendors. XTT-sodium salt, menadione, RPMI powder, and MOPS were purchased from Sigma-Aldrich (St. Louis, MO). Concanavalin A–conjugated with FITC 488 dye was acquired from Thermo Fisher Scientific Inc. (Waltham, MA).

**Table 1:** **MIC of auranofin and control antifungal drugs against Candida and Cryptococcus strains**.

**Strains**	**Description**	**Auranofin (μg/ml)**	**Fluconazole (μg/ml)**	**Flucytosine (μg/ml)**
*C. albicans* NR 29434	Bloodstream isolate from a person with a bloodstream infection collected in Winnipeg, Manitoba, Canada, in 2000	8	4	0.125
*C. albicans* ATCC 10231	Isolated from a man with bronchomycosis	2	2	0.25
*C. albicans* NR 29449	Is a vaginal isolate from a person with vaginitis collected in Ann Arbor, Michigan, USA, between 1990 and 1992	8	2	4
*C. albicans* NR 29435	Is a bloodstream isolate from a person with a bloodstream infection collected in Iowa City, Iowa, USA, in 2000	1	4	0.0625
*C. albicans* NR 29448	Is an isolate from a person with a bloodstream infection, collected in Arizona, USA.	4	>64	0.0625
*C. albicans* NR 29437	Is a bloodstream isolate from a person with a bloodstream infection collected in Brussels, Belgium in 2000	4	2	0.0625
*C. albicans* NR 29446	Is a bloodstream isolate from a person with a bloodstream infection collected in Utah, USA.	16	>64	0.25
*C. albicans* NR 29453	Is an oral isolate from an HIV+ person collected in Pretoria, South Africa	8	2	0.0625
*C. albicans* NR 29438	Is a bloodstream isolate from a person with a bloodstream infection, collected in Tel-Hashomer, Israel, in 2000.	16	2	0.0625
*C. albicans* ATCC 26790	Pulmonary candidiasis	8	2	0.0625
*C. albicans* ATCC 24433	Nail infection	8	4	1
*C. albicans* ATCC 14053	Human blood, Bethesda, MD	8	4	0.125
*C. albicans* ATCC 90028	Blood, Iowa	16	4	1
*C. albicans* NR 29366	Human isolate collected in China	16	>64	0.0625
*C. albicans* NR 29367	Human isolate collected in China.	16	>64	0.0625
*C. glabrata* ATCC MYA-2950	–	8	4	0.0625
*C. glabrata* ATCC 66032	–	8	2	0.0625
*C. tropicalis ATCC 13803*	–	16	2	0.125
*C. tropicalis ATCC 1369*	–	4	1	0.25
*C. parapsilosis ATCC 22019*	Case of sprue, Puerto Rico	4	1	0.25
*C. neoformans* NR-41291	Obtained from human cerebrospinal fluid in China in July 2011.	4	1	0.5
*C. neoformans* NR-41292	Obtained from human cerebrospinal fluid in China in February 2012.	0.5	1	0.5
*C. neoformans* NR-41296	Obtained from human cerebrospinal fluid in China in February 2012.	1	2	0.5
*C. neoformans* NR-41295	Obtained from human cerebrospinal fluid in China in February 2012.	4	2	0.5
*C. neoformans* NR-41294	Obtained from human cerebrospinal fluid in China in June 2011.	0.5	4	2
*C. neoformans* NR-41297	Obtained from human cerebrospinal fluid in China in February 2012.	1	8	4
*C. neoformans* NR-41298	Obtained from human cerebrospinal fluid in China in February 2012.	1	4	2
*C. neoformans* NR-41299	Obtained from human cerebrospinal fluid in China in August 2009.	4	4	2
*C. neoformans* NR-41291	Obtained from human cerebrospinal fluid in China in July 2011.	1	4	1
*Cryptococcus gattii*—CBS1930	Isolated from a goat in Aruba prior to the outbreak in Vancouver, British Columbia, Canada.	0.5	2	2
*Cryptococcus gattii*—R265	Isolated from a human on Vancouver Island, Canada during the outbreak that began in the late 1990's	1	1	1
*Cryptococcus gattii*—Alg40	Progeny of a genotypic cross between *C. gattii* strains R265 and CBS1930.	0.5	2	0.5
*Cryptococcus gattii*—Alg75	Progeny of a genotypic cross between *C. gattii* strains R265 and Alg40.	8	8	8
*Cryptococcus gattii*—Alg81	Progeny of a genotypic cross between *C. gattii* strains R265 and Alg75.	4	8	4
*Cryptococcus gattii*—Alg99	Progeny of a genotypic cross between *C. gattii* strains R265 and Alg81.	4	8	4
*Cryptococcus gattii*—Alg114	Progeny of a genotypic cross between *C. gattii* strains R265 and Alg99.	8	8	4
*Cryptococcus gattii*—Alg115	Progeny of a genotypic cross between *C. gattii* strains R265 and Alg114.	8	8	4
*Cryptococcus gattii*—Alg127	Progeny of a genotypic cross between *C. gattii* strains R265 and Alg115.	4	4	4

### Antifungal susceptibility testing

Antifungal susceptibility testing was carried out as per the National Committee for Clinical Laboratory Standards M-27A3 (NCCLS) guidelines (da Silva et al., 2016). Briefly, the inocula were prepared from 24 h old cultures of *Candida* spp. or 48 h old cultures of *Cryptococcus* spp. in YPD plates. Five colonies were then transferred to 5 mL of sterile 0.9% saline (PBS). The suspensions were adjusted to McFarland standard 0.5 and then diluted 1:2000 in RPMI 1640 buffered to pH 7.0 with 0.165 M MOPS (RPMI-MOPS) to yield an inoculum of 5.0 × 10^2^, −2.5 × 10^3^ CFU/mL. An aliquot (100 μL) of the resulting suspension was incubated with serially diluted fluconazole, flucytosine, and auranofin for 24 h for *Candida* spp and 72 h for *Cryptococcus* spp. The minimum inhibitory concentration (MIC) of fluconazole and flucytosine were determined as the prominent decrease (~50%) in visible growth compared to untreated controls, as per NCCLS guidelines. Similarly the MIC of auranofin was determined as the lowest concentration resulting in 50% reduction in visible growth. All experiments were carried out in triplicate wells.

### Time kill assay

Fungal cultures of *Candida albicans* and *Cryptococcus neoformans* were diluted approximately to 5 × 10^5^ CFU/mL and treated with 5 × and 10 × MICs of auranofin and fluconazole (in triplicate) in RPMI-MOPS, at 35°C. Samples were collected at indicated time points and serially diluted in PBS and plated onto YPD plates. Plates were incubated at 35°C for 24–48 h prior to counting fungal colony forming units (CFU), as described elsewhere (Cantón et al., 2004).

### XTT-reduction assay

*C. albicans* ATCC 10231 was grown in YPD broth at 35°C for 24 h. Cells were washed with PBS and resuspended in RPMI-MOPS at 10^6^ cells/mL (Pierce et al., 2008; Rane et al., 2012). An aliquot (100 μL) of cell suspension was transferred to wells in a 96-well tissue culture plate. After 48 h incubation (at 37°C), wells were washed with PBS and drugs (auranofin, fluconazole, and flucytosine) were added at indicated concentrations. After 24 h of incubation, the supernatant was removed and 100 μL of XTT/menadione solution was added to each well. The plates were covered with aluminum foil and incubated at 37°C for 1 h. Aliquots (75 μL) were taken from each well and the absorbance (OD_495_) was measured using a spectrophotometer. The antifungal activity of each drug was expressed as a percentage of metabolic activity of treatment groups relative to the DMSO-treated control groups. The experiment was performed using triplicate samples for each treatment regimen.

### Confocal imaging of fungal biofilms

*C. albicans* ATCC 10231 was seeded on FBS-coated glass cover slips in 6-well tissue-culture plates and grown in RPMI-MOPS medium with 0.2% glucose at 37°C (Dongari-Bagtzoglou et al., 2009). After 48 h, wells were washed with PBS and drugs (auranofin, fluconazole, and flucytosine) were added at indicated concentrations. After 24 h of treatment, wells were washed with PBS and stained with concanavalin A–conjugated with FITC 488 dye (25 μg/mL in PBS) for 45 min at 37°C. After incubation, the coverslips were washed three times with PBS and mounted on glass slides. Stained biofilms were observed using Leica confocal laser scanning microscopy. Images were reconstructed using IMARIS software.

### Chemogenomics profiling of *Saccharomyces cerevisiae*

Initial testing of *Saccharomyces cerevisiae* sensitivity to auranofin was performed with the wild-type BY4743 diploid strain, the isogenic parent to the heterozygous diploid deletion collection. BY4743 was grown in YPD in 96-well plates with 1% DMSO or auranofin in concentrations ranging from 10 to 200 μM to determine a suitable level of growth inhibition. Auranofin (75 μM) was used for haploinsufficiency profiling because it delayed growth by 30% compared to the no drug control half-maximal optical density (OD). All experiments were performed at 30°C and cultures were shaken at 300 rpm. The heterozygous deletion set was purchased in a pooled format (Thermo Fisher Scientific, Waltham, MA). A frozen aliquot (200 μL) was thawed and used to inoculate 2 mL of YPD and grown for 9 h to reach an OD_600_ of 4.0. The culture was diluted to an OD_600_ of 0.13 and either 1% DMSO or 75 μM auranofin was added (three replicates each, 1 mL) and grown for 7 h. The cultures were grown again by diluting to an OD of 0.13 in 1 mL YPD with DMSO or 60 μM auranofin and grown for 8 h. Cultures were harvested and genomic DNA extracted using the YeaStar Genomic DNA kit (Zymo Research, Irvine, CA). The UPTAGs were amplified by PCR with Phusion Hot Start II High-Fidelity DNA polymerase at 0.02 U/μL (Thermo Fisher Scientific, Waltham, MA) using 0.5 ng/μL genomic DNA. Primers are listed (Table S1). The PCR reactions were electrophoresed on an agarose gel and the 267 bp product extracted using a QIAquick Gel Extraction Kit (Qiagen, Valencia, CA). Purified DNA was measured using a Qubit instrument and samples were normalized and mixed together to a final concentration of 10 nM. Strains were grown and maintained on media according to standard practices (Amberg et al., 2005).

The pooled PCR products were sequenced using standard Illumina sequencing in a HiSeq 2500 instrument. The reads were separated based on a 5 base multiplex tag unique for each experiment and an average of 5 million reads per replicate was obtained. The UPTAG barcodes in each experimental sample were separated based on a reference database of recharacterized barcode sequences (Smith et al., 2009).

The resulting strain counts were imported into R and analyzed with edgeR (Robinson et al., 2010). Sequencing library sizes were normalized using the default parameters. Only strains with one or more counts in three or more samples were analyzed further. Differential representation of strains was determined using the quantile-adjusted conditional maximum likelihood (qCML) method. False discovery rates were determined to control for multiple testing.

### *Saccharomyces* deletion strain haploinsufficiency validation

Overnight grown yeast cells were diluted (OD_600_ ~ 0.03) and grown in the presence and absence of auranofin, at indicated concentrations. Growth was monitored using a spectrophotometer (OD_600_) at indicated time points and the results were expressed as percent growth rate for each strain compared to the untreated control group. To assess growth on solid medium, 5 μL of ten-fold diluted yeast cells were spotted onto YPD agar containing DMSO or auranofin (6.25 μg/mL). Growth of yeast strains was monitored after incubating the plates for 48 h, as described elsewhere (Gamberi et al., 2015).

### Oxygen consumption and membrane potential measurements

Mitochondria were purified from yeast cells grown on YPEG as described previously (Hasson et al., 2010). Oxygen consumption measurements with isolated mitochondria were performed using an oxygen electrode (Hansatec) as described previously (Dabir et al., 2013). Membrane potential measurements of purified mitochondria were performed with fluorescent 3, 3′-dipropylthiadicarbocyanine iodide dye [DiSC3(5)]. 1% DMSO, carbonyl cyanide *m*-chlorophenyl hydrazone (CCCP), MB-6, or MB-7 was added to mitochondria in import buffer (0.6 M sorbitol, 2 mM KH_2_PO_4_, 60 mM KCl, 50 mM HEPES-KOH, 5 mM MgCl_2_, 2.5 mM EDTA, 5 mM L-methionine, pH 7.1) for 10 min. Subsequently 0.2 μM DiSC3(5) in import buffer was added, incubated for 5 min, and fluorescence was measured at excitation and emission length of 620 and 670 nm, respectively.

### Purification of mitochondria

Mitochondria were purified from wild-type yeast or yeast overexpressing Erv1 with a hexahistidine tag ([a2up] Erv1) grown in YPEG as described previously (Glick and Pon, 1995; Dabir et al., 2007). Yeast cultures were kept at 25°C with vigorous shaking during growth. Mitochondria concentration was measured by BCA assay and stored at 25 mg/mL at −80°C. Mitochondria with increased levels of Erv1 were purified from a strain in which Erv1 was overexpressed from a 2-micron plasmid (Dabir et al., 2007).

### Import of radiolabeled proteins into yeast mitochondria

Prior to import into purified mitochondria, [^35^S]-methionine and cysteine labeled proteins were generated with TNT Quick Coupled Transcription/Translation kits (Promega) and plasmids carrying the genes of interest. Transcription of genes was driven by either a T7 or SP6 promoter. Import reactions were conducted as previously described (Hasson et al., 2010; Dabir et al., 2013). After frozen mitochondria aliquots were thawed and added to the import buffer at a final concentration of 100 μg/mL, 1% DMSO or the small molecule was added as indicated. A final concentration of 1% DMSO was used in all experiments. Following incubation at 25°C for 15 min, import reactions were initiated by the addition of 5–10 μl of translation mix. Aliquots were removed at intervals during the reaction time course and import was terminated with addition either of cold buffer or 25 μg/mL trypsin, or the combination. If trypsin was added to digest non-imported precursor protein, soybean trypsin inhibitor was subsequently added in excess after 15 min incubation on ice. After a final recovery of by centrifugation (12,000 × g, 6 min), mitochondria were disrupted in Laemmli sample buffer. Samples from import reaction time points were resolved by SDS-PAGE and visualized by autoradiography. For experiments to investigate the Cmc1-Mia40 intermediate, non-reducing conditions were used. The import reactions were stopped in the presence of 20 mM iodoacetamide and mitochondria disrupted in Laemmli sample buffer lacking β-mercaptoethanol. The imported products were separated by non-reducing SDS-PAGE.

### *Caenorhabditis elegans* (*C. elegans*) infection study

L4-stage worms of *C. elegans* AU37 (sek-1; glp-4) strain (glp-4(bn2) were used to examine the antifungal efficacy of auranofin as described elsewhere (Mylonakis et al., 2002; Thangamani et al., 2015d). Briefly, L4-stage worms were infected with *C. neoformans* NR-41292 for 2 h at room temperature. After infection, worms were washed with M9 buffer and treated either with DMSO or drugs (auranofin, fluconazole, and flucytosine), at a concentration of 8 μg/mL. After 24 h, worms were washed with PBS and disrupted using silicon carbide particles (Thangamani et al., 2015d). The final suspensions were plated onto YPD agar plates containing ampicillin (100 μg/mL), streptomycin (100 μg/mL), and kanamycin (45 μg/mL) to determine the colony forming unit (CFU) per worm (Li et al., 2013).

### Statistical analyses

Statistical analyses were done using GraphPad Prism 6.0 (GraphPad Software, La Jolla, CA). *P* values were calculated via the Student *t*-test and *P*-values of ≤0.05 were deemed significant.

## Results

### Antifungal activity and killing kinetics of auranofin

The antifungal activity of auranofin was tested against various clinical isolates of *Candida* and *Cryptococcus*. Auranofin was very active in inhibiting the growth of all strains of *C. albicans, C. glabrata, C. tropicalis*, and *C. parapsilosis* with the MIC ranging from 1 to 16 μg/ml (Table 1). Auranofin also displayed potent activity against both *C. neoformans* and *Cryptococcus gattii* inhibiting growth of these fungal species at a concentration ranging from 0.5 to 8 μg/ml (Table 1).

**Figure 1:**
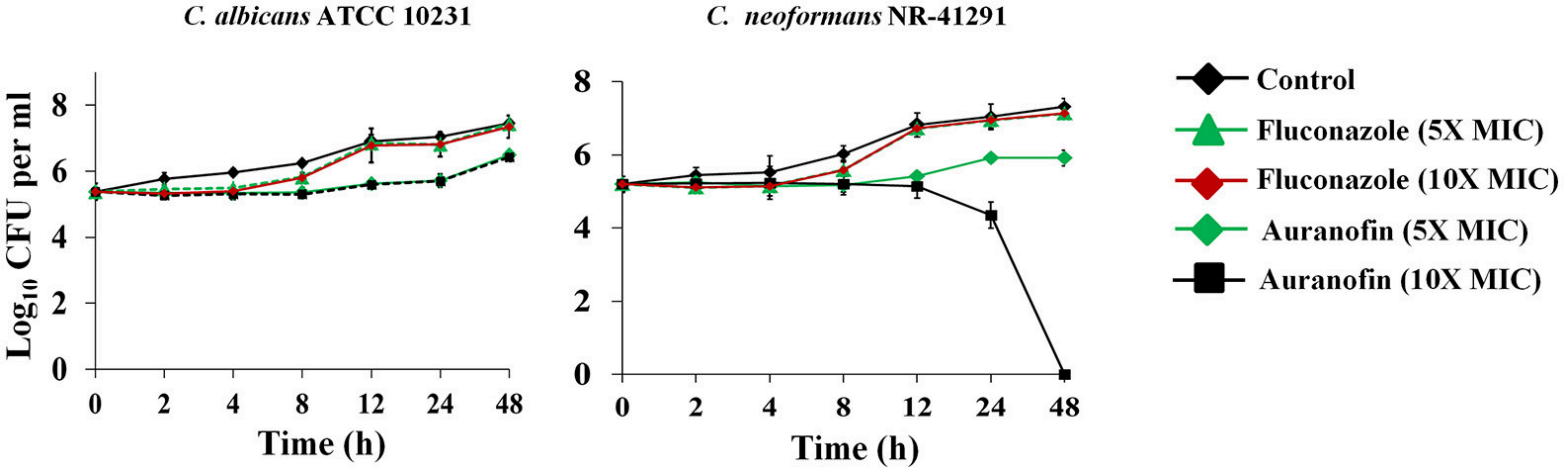
**Killing kinetics of auranofin**. An overnight culture of *C. albicans* ATCC 10231 and *C. neoformans* NR-41291 were treated with 5 × and 10 × MIC of auranofin and fluconazole (in triplicate) in RPMI-MOPS and incubated at 35°C. Samples were collected at indicated time points and plated onto YPD plates. Plates were incubated for 24–48 h prior to counting the colony forming units (CFU).

A time-kill assay was employed to investigate the killing kinetics of auranofin against both *C. albicans* and *C. neoformans*. Similar to fluconazole, auranofin (at the 5 × MIC) exhibited fungistatic activity against *C. albicans* ATCC 10231 and *C. neoformans* NR-41296 (Figure 1). However, at the 10 × MIC, auranofin and fluconazole exhibited fungistatic activity against *C. albicans* ATCC 10231, whereas unlike fluconazole, auranofin (at the 10 × MIC) completely kills *C. neoformans* NR-41296 after 48 h of incubation (Figure 1).

### Antibiofilm activity of auranofin

**Figure 2:**
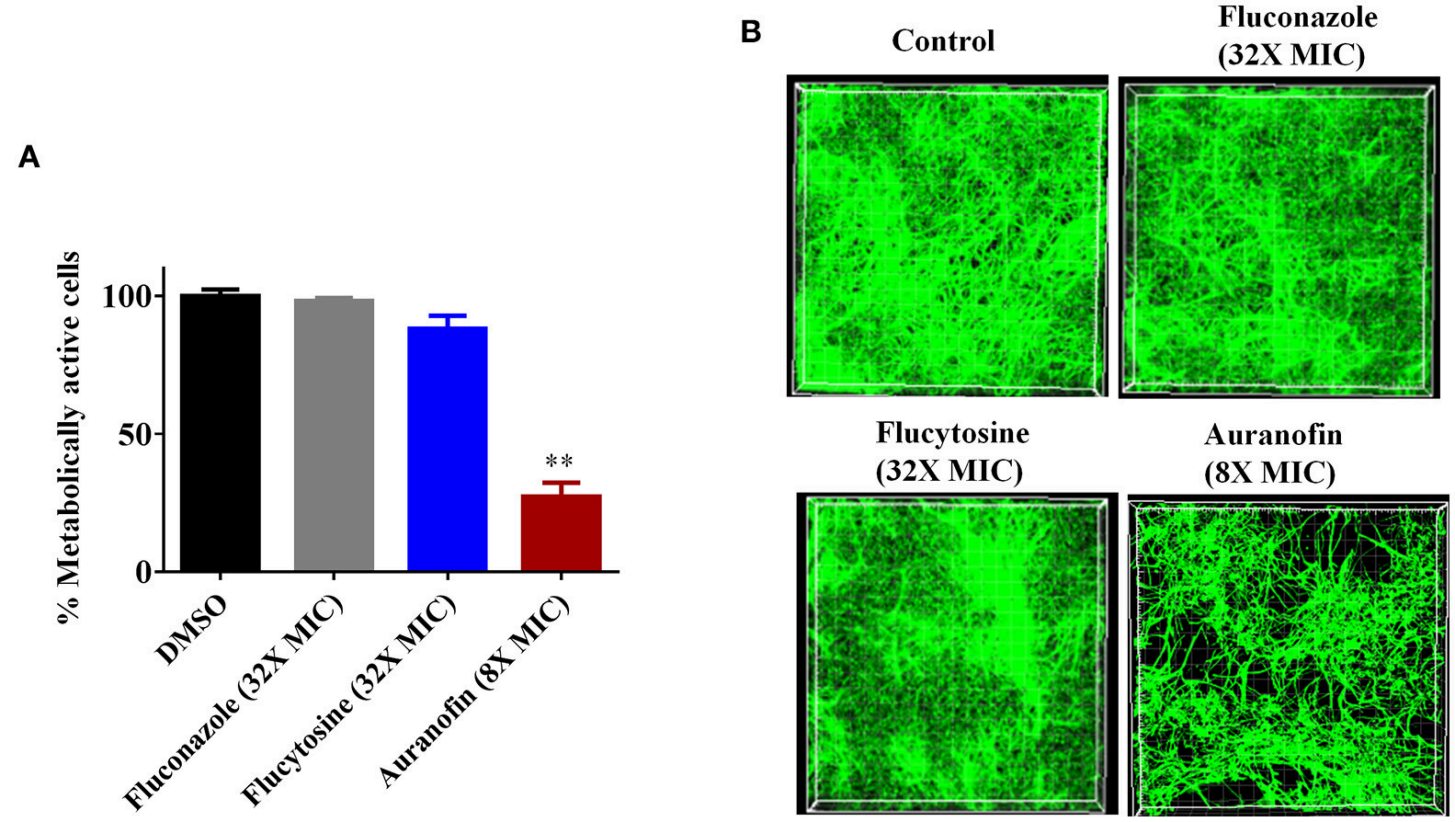
**Effect of auranofin on *Candida* biofilms. (A)** *C. albicans* ATCC 10231 biofilm was treated with indicated concentrations of auranofin, fluconazole, and flucytosine for 24 h. The percent metabolic activity of fungal cells in biofilms, after treatment, was determined using the XTT reduction assay. Results are presented as means ± *SD* (*n* = 3). Statistical analysis was calculated using the two-tailed Student's *t*-test. *P*-values (^**^P ≤ 0.01) are considered as significant. Auranofin was compared both to controls and antifungal drugs (^**^). **(B)** *C. albicans* ATCC 10231 biofilm was formed on FBS-coated glass cover slips and treated with indicated drugs for 24 h and stained with concanavalin A– conjugated with FITC dye and imaged by Leica confocal laser scanning microscopy.

The antibiofilm activity of auranofin, against *C. albicans*, was evaluated using the XTT reduction assay in order to measure the metabolic activity of fungal cells post-treatment. The metabolic activity of *C. albicans* was reduced by more than 70% with the treatment of auranofin at 8 × MIC (Figure 2A). However, the control antifungals fluconazole and flucytosine were ineffective (less than 10% reduction observed) at reducing metabolic activity of *C. albicans* biofilm, even at a concertation equivalent to 32 × MIC when compared to the DMSO-treated control groups (Figure 2A).

The effect of auranofin on reducing fungal biofilm density was further evaluated using confocal microscopy. Fungal cells stained with ConA conjugated with FITC revealed that auranofin (8 × MIC) eradicates a considerable portion of *Candida* cells in comparison to the control group (Figure 2B). However, treatment with fluconazole and flucytosine, even at 32 × MIC, appear similar to control group (Figure 2B). These results correlate with the results of the XTT reduction assay.

### Chemogenomic profiling identifies Mia40 as a potential target of auranofin

**Figure 3:**
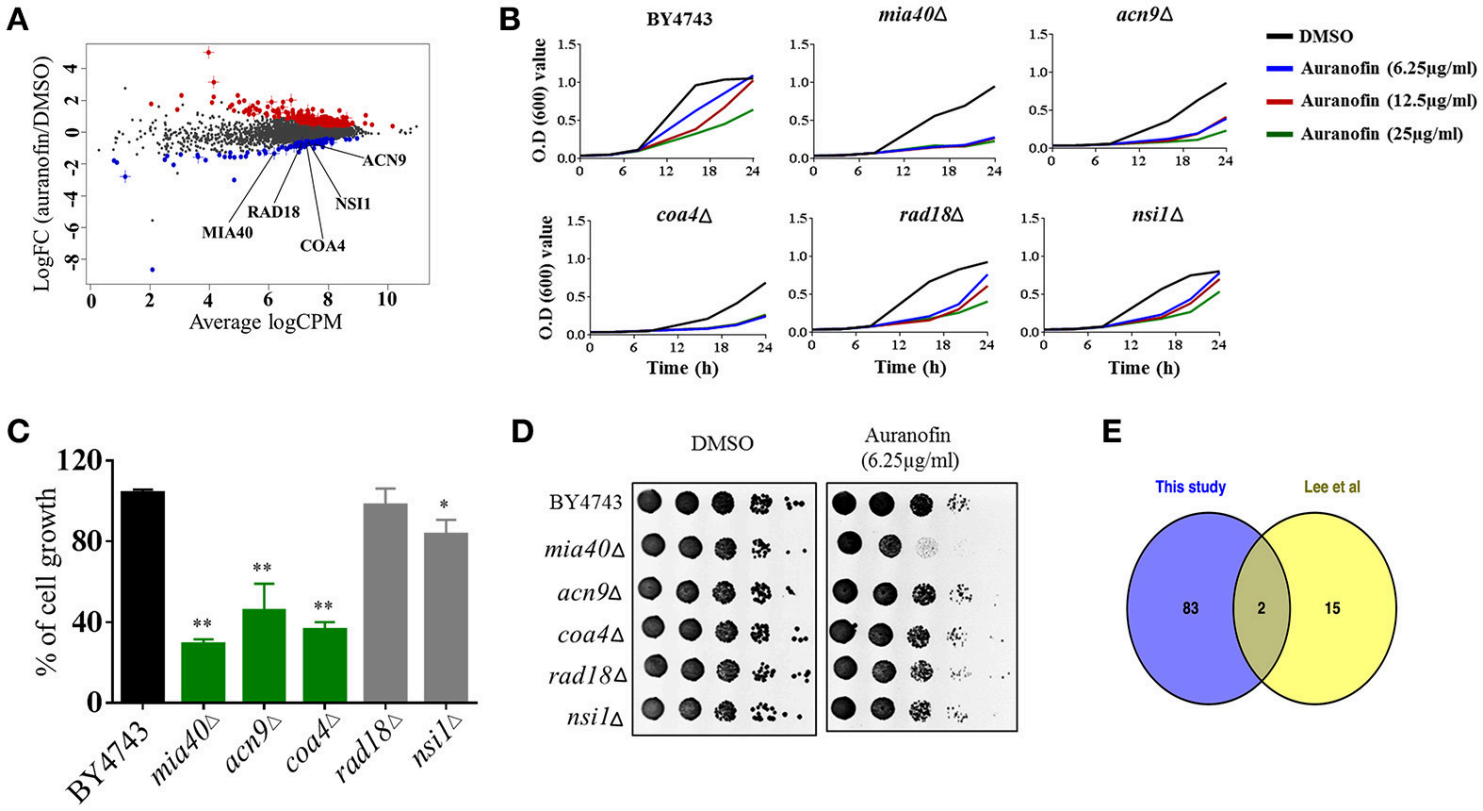
**Auranofin targets mitochondrial protein(s). (A)** Chemogenomic profiling of *S. cerevisiae* with treatment of auranofin. The strain abundance were normalized using EdgeR and shown. **(B)** Growth curve of wild type (BY4743) and heterozygous deletion strains (*mia40*Δ, *acn9*Δ, *coa4*Δ, *rad18*Δ, and *nsi1*Δ) in the presence of indicated concentration of auranofin in YPD broth were determined. **(C)** The percent growth of yeast cells (OD_600_ after 24 h) incubated with auranofin (6.25 μg/mL) in YPD broth was determined in relation to the DMSO treatment. The results are presented as means ± *SD* (*n* = 3). Statistical analysis was calculated using the two-tailed Student's *t*-test. *P*-values (^*^P ≤ 0.05) (^**^P ≤ 0.01) are considered as significant. **(D)** Yeast cells grown in YPD broth overnight were serially diluted and spotted on solid YPD agar containing auranofin (6.25 μg/mL) or DMSO and the CFU were shown. **(E)** Comparison of Lee et al. (2014) HIP results with our 85 strains are shown as a Venn diagram.

To investigate the MOA, we subjected auranofin to chemogenomic profiling using the *S. cerevisiae* heterozygous deletion collection. Haploinsufficiency profiling (HIP) allows for the simultaneous assessment of the sensitivity of the pooled genome-wide set of heterozygous deletion strains because each strain is uniquely identified with a synthetic DNA barcode. The method is an unbiased approach to survey the genome-wide strain set in order to identify the strains with the most sensitivity to auranofin. We first identified the concentration that reduced wild-type growth by 30% and used 75 μM to profile the pooled heterozygous strains in biological samples. PCR was used to amplify the unique UPTAG DNA barcodes located at the gene deletion site and we tracked the barcode abundance with Illumina sequencing. The resulting counts were normalized and visualized using EdgeR (Figure 3A). We identified 85 heterozygous deletion strains that were under-represented based on an FDR ≤ 0.1 when comparing auranofin treatment to DMSO. These 85 strains were analyzed to identify associated gene ontology cellular component annotations and found to be enriched in several categories including the mitochondrial intermembrane space and chromatin components (Supplementary data excel file). Five heterozygous deletion strains within these enriched categories (*mia40*Δ, *acn9*Δ, *coa4*Δ, *rad18*Δ, and *nsi1*Δ) were selected to validate sensitivity to auranofin using a variety of growth assays (Figure 3A). These strains were randomly selected on the basis that they represented genes in the highest significance gene ontology categories including “regulation of translational initiation” (*p*-value = 0.00033) and “mitochondrial intermembrane space” (*p*-value = 0.0072) (Supplementary data excel file). In addition, as outlined in the following section, the *mia40*Δ strain was tested because it was implicated as sensitive to auranofin in a previous study (Lee et al., 2014).

Growth of these five heterozygous deletion strains and the wild-type (BY4743) strain were monitored in the presence of different concentrations of auranofin (6.25, 12.5, and 25 μg/mL) in a liquid growth assay. The result indicated that only three heterozygous deletion strains (*mia40*Δ, *acn9*Δ, and *coa4*Δ) exhibited drug-induced haploinsufficiency under these conditions. The growth of these deletion strains was suppressed, even in the presence of low concentrations of auranofin (6.25 μg/mL) (Figure 3B). However, auranofin does not induce haploinsufficiency in the other two deletion strains (*rad18*Δ and *nsi1*Δ) as growth of these strains, in the presence of auranofin, mimics the pattern observed with the wild-type strain (Figure 3B). These two deletion strains were not affected possibly because of the concentration used in our validation studies or because they may be false positives. For each strain, the growth of cells (OD_600_ after 24 h) incubated with auranofin (6.25 μg/mL) was determined in relation to DMSO treatment. The growth of three heterozygous deletion strains (*mia40*Δ, *acn9*Δ, and *coa4*Δ) was drastically suppressed by more than 50% in the presence of auranofin (6.25 μg/mL). However, the remaining two deletion strains (*rad18*Δ and *nsi1*Δ) had a modest reduction in growth of~10% compared to the wild-type strain (Figure 3C).

The growth of these five deletion strains was further confirmed by spotting serial dilutions of cultures on solid agar. As shown in Figure 3D, growth of the wild-type and five heterozygous deletion strains was normal in agar containing DMSO. However, the heterozygous deletion strain, *mia40*Δ, exhibited a nearly two-fold reduction in colony forming units when spotted onto YPD agar containing auranofin (6.25 μg/mL).

A study conducted by Lee et al. (2014) previously analyzed a heterozygous deletion pool, representing essential genes, using haploinsufficiency profiling with hundreds of compounds including auranofin. Examination of theauranofin-generated data set shows that they identified 17 strains as possibly sensitive. Comparison of Lee et al.'s results with our 85 strains showed that two strains, *rho1*Δ and *mia40*Δ, overlapped between the data sets (Figure 3E). An additional study by Gamberi et al. (2015) specifically assessed sensitivity and resistance of haploid deletion strains involved in mitochondrial function and found them to be differentially effects to auranofin compared to the haploid wild type parental strain. Based on studies by Gamberi et al. (2015) and Lee et al. (2014), we next moved to examine sensitivity of the corresponding heterozygous deletion strains involved in mitochondrial function and redox homeostasis that are possibly sensitive to auranofin.

**Figure 4:**
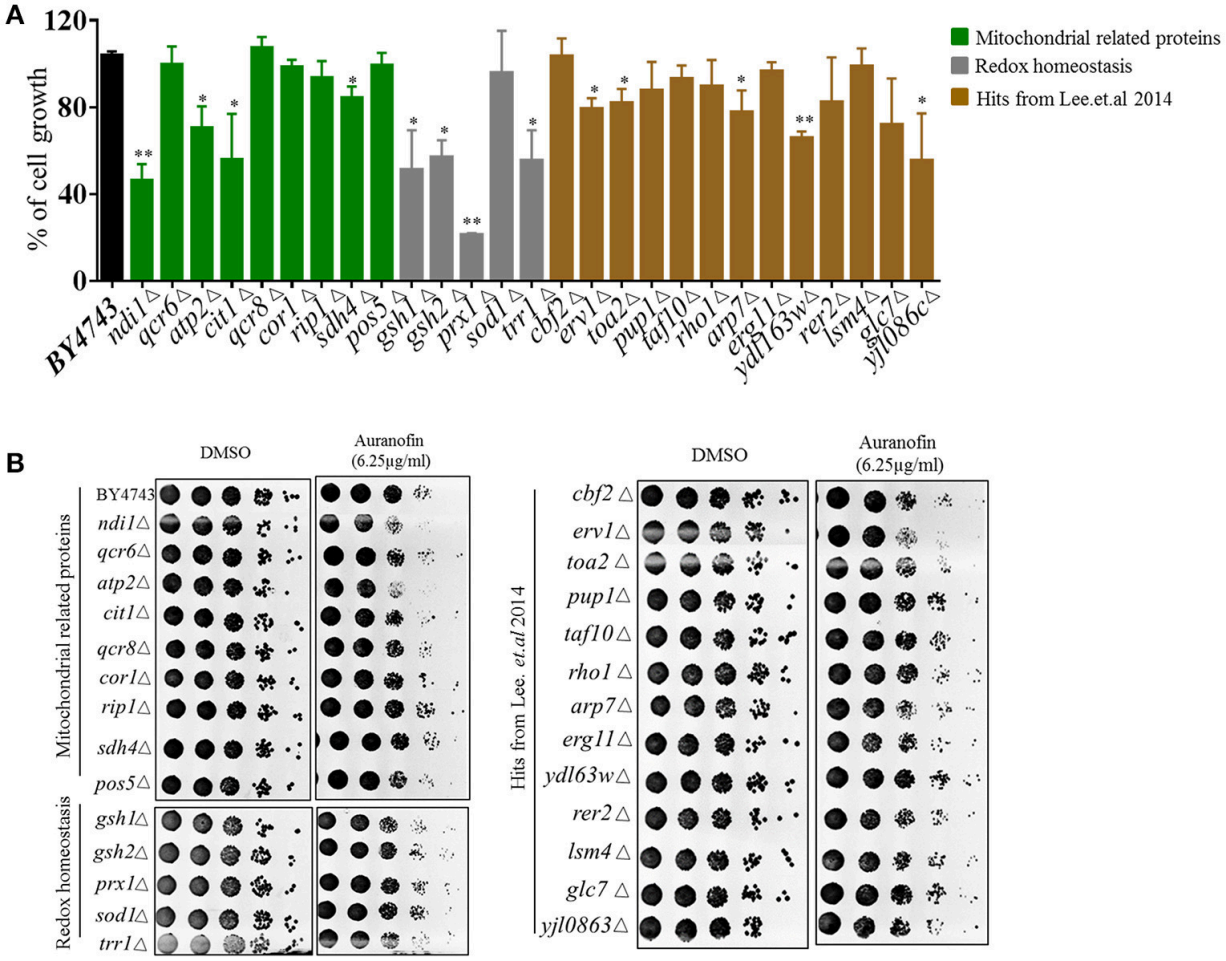
**Effect of auranofin on deletion strains related to ROS production and mitochondrial function**. **(A)** The percent growth of wild type and heterozygous deletion strains incubated with auranofin (6.25 μg/mL) in YPD broth (OD_600_ after 24 h) was determined in relation to the DMSO treatment. The results are presented as means ± *SD* (*n* = 3). Statistical analysis was calculated using the two-tailed Student's *t*-test. *P*-values (^*^P ≤ 0.05) (^**^P ≤ 0.01) are considered as significant. **(B)** Yeast cells grown in YPD broth overnight were spotted on solid YPD agar containing auranofin (6.25 μg/mL) or DMSO. The colony forming units are shown.

Heterozygous deletion strains with genes deleted in mitochondrial function and redox homeostasis experienced a significant growth reduction when treated with auranofin (6.25 μg/mL) relative to DMSO-treated cells (Figure 4A–green and gray bars), including *ndil*Δ*, atp2*Δ*, citl*Δ*, sdh4*Δ*, gsh1*Δ*, gsh2*Δ*, prx1*Δ*, trr1*Δ, *erv1*Δ*, toa2*Δ*, arp7*Δ*, ydl63w*Δ*, and yjl086c*Δ. These results are in agreement with Gamberi's et al. (2015) results in that mitochondrial function appears to be a target of auranofin. It should be noted that Gamberi et al. used haploid deletion strains sensitivity, which generally does not inform on the direct target of a compound as opposed to the heterozygous deletion strains used in our study. In addition, Gamberi et al. examined resistance of haploid deletion strains and varied media conditions, resulting in their conclusion that Pos5 was the target—however, several other haploid deletions strains also demonstrated slight resistance to auranofin and heterozygous strain sensitivity was not examined. Our results demonstrate that the heterozygous *pos5*Δ strain is not sensitive to auranofin in liquid or agar conditions suggesting Pos5 is unlikely to be the direct target of auranofin (Figures 4A,B). The heterozygous strains that were identified by Lee et al. (2014) were generally not as sensitive in liquid growth compared to mitochondrial or redox-related strains (Figure 4A—brown bars). Because this set of strains contained deletions of genes with a wide variety of functions it is likely that they not specific hits from the screen and shows the importance of testing individual strains to confirm sensitivity to auranofin. These results were confirmed using the YPD agar-spotting assay in that many strains were not sensitive. Interestingly, heterozygous deletion strains involved in ROS response and redox homeostasis (*sdh4*Δ*, gsh1*Δ*, gsh2*Δ, and *prx1*Δ) which had significant growth reduction in liquid medium did not demonstrate considerable reduction in growth when spotted onto YPD agar containing auranofin (6.25 μg/mL) (Figure 4B).

As noted earlier, heterozygous deletion strains that encode genes required for mitochondrial function (including *ndil*Δ*, atp2*Δ*, citl*Δ, and *erv1*Δ), showed a considerable decrease in colony count (almost one-fold log reduction) when spotted onto YPD agar containing auranofin (6.25 μg/mL) (Figure 4B). Interestingly, a deletion strain (*erv1*Δ), which forms a complex with Mia40 (Rissler et al., 2005), showed considerable sensitivity to auranofin, which coincides with Lee et al.'s findings (Figure 4B). Taken altogether, our results as well as the overlap with the previous haploinsufficiency profiling (Lee et al., 2014), supports the notion that Mia40/Erv1 is the probable antifungal target of auranofin.

### Auranofin inhibits the import of Mia40 substrate (Cmc1)

**Figure 5:**
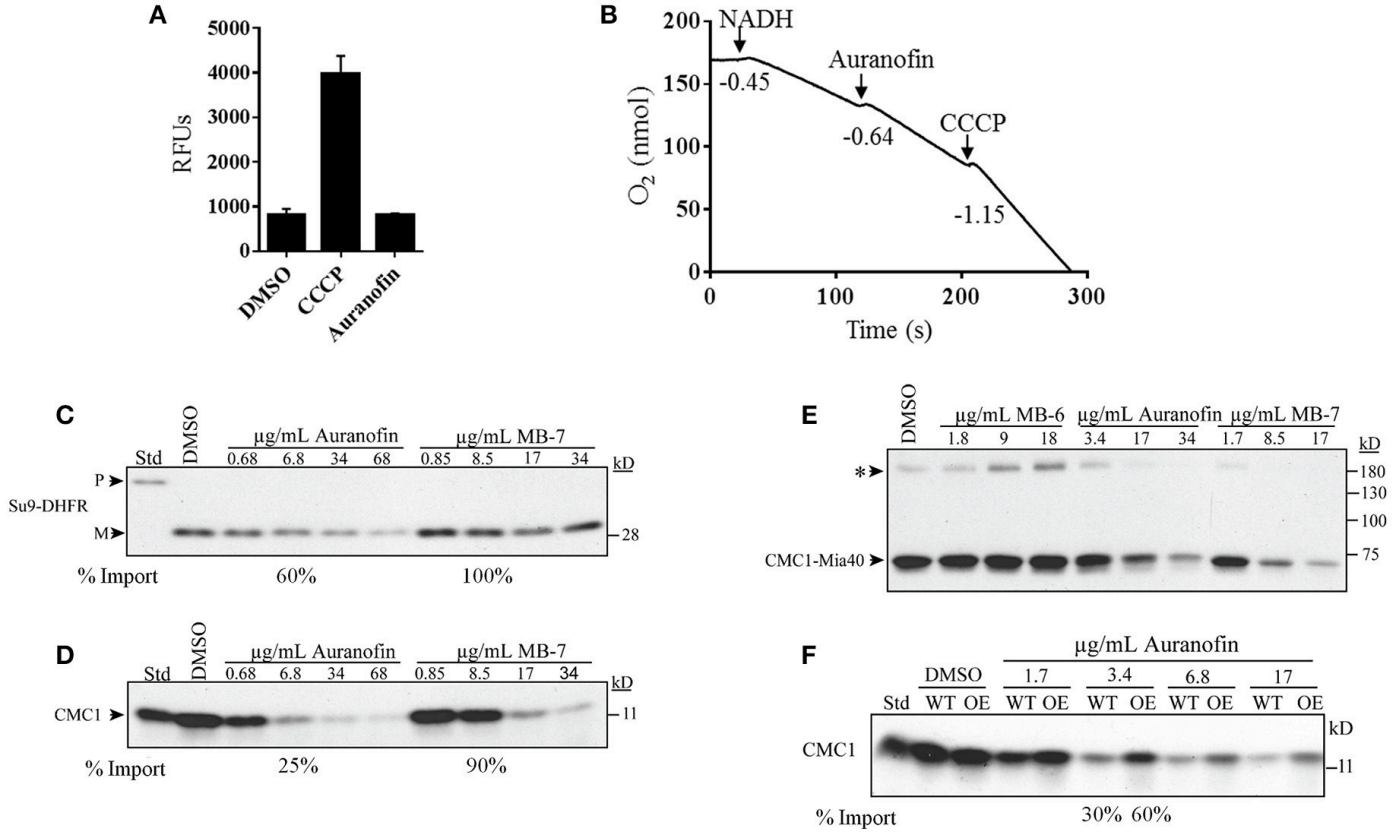
**Auranofin does not impair general mitochondrial function but inhibits the import of substrates of the Mia40 pathway. (A)** Mitochondrial uptake and quenching of DiSC3(5) dye when membrane potential is present. Dye fluorescence was measured as relative fluorescence units (RFUs) in the presence of DMSO, auranofin, and CCCP. **(B)** Respiration of mitochondria was initiated by NADH followed by the addition of auranofin and CCCP. Respiration levels measurements were performed using an oxygen electrode and rates represent the consumption of O_2_ nmol/s. **(C,D)** Radiolabeled proteins Su9-DHFR and Cmc1 were imported into mitochondria in the presence of varying concentrations of auranofin and MB-7. **(E)** Non-reducing gel demonstrating the formation of the Cmc1-Mia40 intermediate in the presence of auranofin, MB-6 and MB-7. **(F)** Auranofin inhibition of protein import is dependent on *in organello* mitochondrial Erv1 expression level. Wild-type (WT) and Erv1 overexpressed (OE) mitochondria were treated with varying concentrations of auranofin and the level of radiolabeled Cmc1 was detected. The asterisk represents a large complex of unknown composition that is observed in most Mia40 precursor studies. Representative gels have been shown (*n* = 3).

To further confirm the specific inhibition of the Mia40-Erv1 pathway by auranofin we employed several biochemical experiments using purified yeast mitochondria similar to a previous study that investigated the effect of several small molecule inhibitors of redox-regulated protein import into mitochondria (Dabir et al., 2013). A possible indirect mechanism of inhibition of mitochondrial function and the Mia40-Erv1 pathway is by the disruption of membrane potential or diminished oxidative phosphorylation. Maintenance of membrane potential was determined by mitochondrial uptake of DiSC3 (5) dye and subsequent quenching in the presence of membrane potential. Auranofin had no effect on the membrane potential compared to DMSO whereas the uncoupling agent, CCCP, caused a 4-fold increase in fluorescence indicating uncoupled mitochondria (Figure 5A). The effect on mitochondrial respiration was determined by measuring dissolved oxygen in a chamber with purified mitochondria and respiration was initiated with NADH resulting in an oxygen consumption rate (−0.45 O_2_ nmol/s) consistent with well-coupled mitochondria. The addition of DMSO did not increase respiration rate and auranofin at a concentration of 34 μg/mL only slightly increased the respiration rate (−0.64 O_2_ nmol/s) (Figure 5B and Table S2). As a control, the addition of CCCP resulted in a severe increase in consumption rate (−1.15 O_2_ nmol/s) suggestive of uncoupled mitochondria (Figure 5B).

To confirm that auranofin targets the Erv1/Mia40 pathway we measured the effect of compound on the import of mitochondrial protein substrates compared to control compounds previously identified as Erv1 inhibitors (Dabir et al., 2013). Radiolabeled precursor proteins were incubated with mitochondria in the presence of small molecules or DMSO and the reaction was terminated with protease and subsequently analyzed by gel electrophoresis. Protein substrates from different import pathways were assessed including the Tim23 substrate, Su9-DHFR, and the Mia40 substrate, Cmc1. Auranofin at a lower concentration of 6.8 μg/mL inhibits import of Su-DHFR to a 60% level and Cmc1 to a 25% level compared to untreated samples (Figures 5C,D). These results indicate the preferential activity of auranofin toward inhibiting Cmc1 import compared to Su9-DHFR, which is expected because Cmc1 is directly imported by Mia40/Erv1. Strikingly, auranofin exhibits more potent activity than control compound, MB-7 with a drastic difference in import efficiency observed between the compounds at 10 μM (6.8 μg/mL for auranofin and 8.5 μg/mL for MB-7; Figure 5D). Although auranofin does inhibit Su9-DHFR import at high concentrations, these results demonstrate the compound has specificity toward the Mia40 pathway and increased potency compared to previously identified inhibitors from a large-scale chemical library screen (Dabir et al., 2013). It is not surprising that the import of Su9-DHFR is mildly inhibited because mitochondrial import pathways are interconnected.

Mia40 has previously been demonstrated to form an intermediate with Cmc1 as part of the import process (Bourens et al., 2012; Neal et al., 2015). The effect of compounds on the formation of a disulfide intermediate between Mia40 and Cmc1 was monitored *in organello*. Auranofin inhibits radiolabeled Cmc1 from interacting with Mia40 in a similar dose dependent manner to MB-7 (Figure 5E). The addition of another control, MB-6, causes the accumulation of the intermediate. In sum, auranofin inhibits the heterodimer formation of the Mia40-Cmc1 intermediate and is a potent inhibitor of the Mia40 import pathway.

### Overexpression of Erv1 in yeast mitochondria confers resistance to auranofin

To further validate the Mia40 pathway as a target of auranofin, import of Cmc1 was performed with mitochondria from WT and Erv1 overexpressing yeast. Erv1 overexpression is expected to maintain the Mia40 pool in an oxidized state, which is required for the interaction with substrate proteins (Mesecke et al., 2005; Dabir et al., 2007) and hence should be more resistant to auranofin inhibition. As predicted, the Erv1 overexpressing mitochondria were resistant to auranofin (3.4 μg/mL) treatment as evidenced by the increased level of Cmc1 (60%) import compared to WT (30%) mitochondria providing further confirmation of Mia40 as a target (Figure 5F).

### *In vivo* efficacy of auranofin in *C. neoformans* infected *C. elegans* model

**Figure 6:**
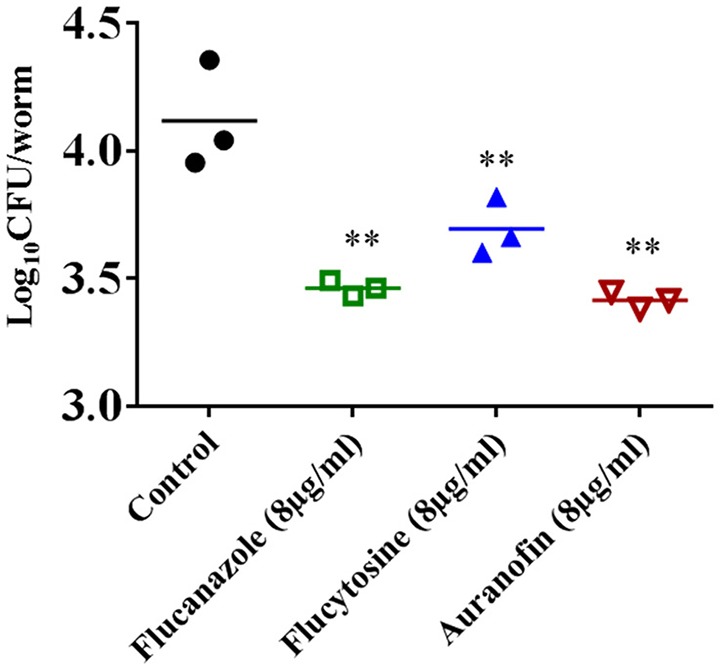
**Efficacy of auranofin in *C. neoformans*-infected *C. elegans***. L4-stage worms were infected with *C. neoformans* and treated with auranofin, fluconazole, and flucytosine, at a concentration of 8 μg/mL. After 24 h, worms were lysed and plated onto YPD plates to determine the CFU per worm. Each dot represents average fungal load in each worm per well. The results are presented as means ± *SD* (*n* = 3). Statistical analysis was calculated using the two-tailed Student's *t*-test. *P*-value (^**^P ≤ 0.01) are considered as significant.

To investigate if the *in vitro* antifungal activity of auranofin translates *in vivo*, the antifungal efficacy of auranofin was examined in a *C. neoformans*-infected *C. elegans* animal model. As shown in Figure 6, treatment of infected *C. elegans* with fluconazole, flucytosine, and auranofin, at 8 μg/mL, produced a significant reduction (*P* ≤ 0.01) in mean fungal load when compared to the untreated control groups. Strikingly, *C. elegans* treated with auranofin (8 μg/mL) generated the largest reduction in *C. neoformans* CFU (0.87 ± 0.03 log_10_), followed by fluconazole (8 μg/mL) (0.82 ± 0.03 log_10_) and flucytosine (8 μg/mL) (0.58 ± 0.11 log_10_) (Figure 6).

## Discussion

Auranofin has a well-established pharmacological and toxicological profile that has permitted it to be used for the treatment of rheumatoid arthritis for more than 30 years (Bernhard, 1982; Furst et al., 1983). Independent of its antirheumatic effect, several studies have reported the anti-infective properties of this drug against important parasitic and bacterial pathogens including *Schistosoma mansoni, Trypanosoma brucei, Plasmodium falciparum, Entamoeba histolytica, Staphylococcus aureus*, and *Streptococcus pneumoniae* (Jackson-Rosario et al., 2009; Debnath et al., 2012; Cassetta et al., 2014; Harbut et al., 2015; Thangamani et al., 2016a,b). However, a dichotomy exists regarding the antimicrobial MOA of auranofin. Debnath et al. (2012) and Harbut et al. (2015) reported that auranofin exhibits its antimicrobial activity through the inhibition of the thioredoxin reductase (TrxR) enzyme in both *E. histolytica* and *S. aureus*. However, a recent crystallographic study conducted by Parsonage et al. (2016) revealed that auranofin most likely does not bind to the cysteine residues in TrxR of *E. histolytica*. Another study, conducted by our research group, also demonstrated that TrxR is not the primary target of auranofin in bacteria (Thangamani et al., 2016a). We demonstrated that auranofin inhibits multiple biosynthetic pathways including DNA, protein and cell wall synthesis in bacteria (Thangamani et al., 2016a). However, the exact molecular target of auranofin, in bacteria, still remains unclear.

The FDA's approval of auranofin as an anti-amoebic agent opened the door for researchers to examine additional clinical applications for this drug (Debnath et al., 2012). Recent studies, including the present work, demonstrate that auranofin inhibits the planktonic growth of multiple species of fungi including *C. albicans, C. glabrata, C. tropicalis, C. parapsilosis, C. neoformans*, and *C. gattii with* an average MIC of 4 μg/ml (Stylianou et al., 2014; Fuchs et al., 2016).

In addition to planktonic growth, fungi especially, *Candida* spp., are known to form biofilms that are recalcitrant to treatment with antifungal agents. Fungal cells encased within the biofilm are resistant to most clinically used antifungals, including azole drugs, ultimately resulting in treatment failure (Chandra et al., 2001; Mathé and Van Dijck, 2013; Chandra and Mukherjee, 2015). Biofilm-related *C. albicans* infections thus pose a major threat to the public (Mathé and Van Dijck, 2013). Therefore, we examined the effect of auranofin on adherent *C. albicans* biofilms. Our results indicate that auranofin is very effective in disrupting *C. albicans* biofilm as this drug is able to reduce the metabolic activity of fungal cells present within the biofilm by more than 75% (relative to the control groups). Further examination of *C. albicans* biofilm using confocal microscopy revealed that auranofin treatment markedly reduces the number of fungal cells present in the biofilm compared to the control and treatment groups (fluconazole and flucytosine). These findings illustrate that auranofin is a potential candidate for use in treatment of biofilm-related fungal infections.

After verifying auranofin's antifungal activity, we proceeded to investigate the antifungal mechanism of auranofin. To examine auranofin's antifungal MOA, chemogenomic profiling was employed given it is a highly-specific technique to investigate the target of unknown compounds (Giaever et al., 2004; Roemer et al., 2012). This technique uses drug-induced haploinsufficiency, where it causes a strain-specific fitness defect after treatment with compounds, and thereby aids in identifying the drug target (Giaever et al., 2004; Roemer et al., 2012). In our study, chemogenomic profiling of *S. cerevisiae* with auranofin identified three heterozygous deletion strains of genes (*mia40*Δ, *acn9*Δ, and *coa4*Δ) involved in mitochondrial function were found to be highly susceptible to auranofin. On the other hand, genes (*rad18*Δ *and nsi1*Δ) involved in chromatin function were found to not be affected possibly because of the concentration used in our validation studies or because they may be false positives from the profiling data. Our results are in agreement with Gamberi et al. (2015) indicating the mitochondrial protein(s) is the potential target of auranofin. We further confirmed the three heterozygous deletion strains (*mia40*Δ, *acn9*Δ, and *coa4*Δ) were sensitive to auranofin when grown in YPD liquid medium with auranofin. Interestingly, only one deletion strain, *mia40*Δ, was very sensitive to auranofin in YPD agar containing the drug. Gamberi et al. demonstrated that another mitochondrial protein, the Pos5 NADH kinase, is thought to be the likely target of auranofin because the haploid deletion strain exhibited resistance. However, our chemogenomic profiling results did not identify *pos5*Δ heterozygous deletion strain as sensitive. When the *pos5*Δ heterozygous deletion strain was examined in our study, it was found to be not sensitive to auranofin. Chemogenomic profiling by Lee et al. also did not identify the *pos5*Δ heterozygous deletion strain as sensitive to auranofin (Lee et al., 2014). We also examined additional genes reported in Gamberi et al.'s study and found heterozygous deletion strains involved in mitochondrial function (*ndil*Δ*, and atp2*Δ) showed moderate susceptibility to auranofin. This aligns with the results of Gamberi et al. where they observed resistance to auranofin in haploid deletion strains for these two genes (Gamberi et al., 2015).

Chemogenomic profiling of 6000 genes in *S. cerevisiae* is a non-biased technique and further the gene (mia40) identified by this method also overlaps with data from Lee et al. (2014). Auranofin is one of 300 compounds screened using haploinsufficiency profiling and results from Lee et al.'s study indicates that mia40 is one of the target genes for auranofin (Lee et al., 2014). In addition, we also observed that another deletion strain (*erv1*Δ), which forms a complex with mia40 as reported in Lee et al.'s study, also showed considerable sensitivity to auranofin (Rissler et al., 2005).

The Mia40 (mitochondrial intermembrane space import and assembly protein 40)–Erv1 pathway is mainly involved in oxidation of several cysteine rich proteins that enter the mitochondria from the cytoplasm (Rissler et al., 2005; Banci et al., 2009). These proteins, present in the inner mitochondrial space, are essential for cell viability and are functionally linked to the respiratory chain (Rissler et al., 2005; Bihlmaier et al., 2007). In addition, an *ERV1* mutant strain was shown to be deficient in respiration (Lisowsky, 1992) consistent with the metabolic shift from respiration to fermentation observed in auranofin treated cells (Gamberi et al., 2015). Results from the present study indicates that auranofin does not have a generalized mode of action resulting in the disruption of membrane potential or respiration and mitochondrial integrity is maintained in the presence of the compound. However, auranofin preferentially inhibits the import of mitochondrial protein substrate Cmc1 which is directly imported by Mia40/Erv1pathway. Also, overexpression of Erv1confered resistance to auranofin which is directly noticed by the increased level of Cmc1 import. Taken together, our findings support the notion that auranofin preferentially targets Mia40/Erv1pathway in yeast. It should also be taken into account the affinity of auranofin to human Mia40 protein. The central part of the human homolog of Mia40 shares high sequence identity with most of its eukaryotic analog. However, Mia40 in yeast differs from its human homolog in one major respect—yeast Mia40 lacks the N-terminal extension including a transmembrane region (Banci et al., 2009). Future studies are needed to examine the affinity and binding of auranofin to human Mia40 protein. It may be possible that a therapeutic window exists because human Mia40 is not accessible or affected by auranofin at the concentrations needed for antifungal activity.

As reported earlier, in bacteria and parasites the thioredoxin reductase gene was proposed to be the target of auranofin (Debnath et al., 2012). Gamberi et al. used homozygous deletion strains and demonstrated that auranofin does not displayed resistance to both the mitochondrial thioredoxin reductase (*TRR2*) and glutathione reductase (*GLR1*) genes in *S. cerevisiae* (Gamberi et al., 2015). However, the effect of auranofin on cytoplasmic thioredoxin reductase (*TRR1*) gene was not explored in that study (Gamberi et al., 2015). Results from our investigation indicate that the heterozygous deletion strain (*trr1*Δ) behaves similar to wild type. We therefore conclude that auranofin does not target the thioredoxin reductase gene in yeast which is in agreement with a previous study (Gamberi et al., 2015).

The genes involved in ROS response (*SOD1, GSH1, GSH2*, and *PRX1*) were also examined in this study. Our results revealed that heterozygous deletion strains encoding these genes were sensitive to auranofin when grown in YPD liquid broth but not in YPD agar containing auranofin. Gamberi et al. through various experiments demonstrated that auranofin does not elicit the production of ROS (Gamberi et al., 2015) but some haploid deletion strains were sensitive suggesting they are selectively important for resistance to auranofin. Taken together it appears that the ROS response enzymes are not direct targets but some enzymes do mediate resistance to inhibitory activity by auranofin that is not due to generation of ROS.

The final step in our study involved investigating the *in vivo* efficacy of auranofin in a *C. neoformans*-infected *C. elegans* animal model. Auranofin significantly reduced the mean fungal load in worms compared to control groups. Future studies are needed to test the efficacy of auranofin in an appropriate mouse model of fungal infection. Altogether, results from our study suggests that auranofin, with its unique MOA and potent *in vivo* antifungal activity, warrants further investigation as an antifungal agent to combat drug-resistant fungal infections. Auranofin has advantageous qualities to be repurposed as an antifungal agent, including oral bioavailability, clinically safe, potent broad-spectrum fungicidal activity, and the ability to cross blood brain barrier. The characteristics of auranofin as an antifungal agent offer a significant improvement over current approaches for treating fungal infections and provide valuable evidence that auranofin has significant promise to be repurposed as a novel antifungal drug.

## Author contributions

ST, MM, and LA performed the experiments. PP analyzed the sequencing results. ST, TH, MM, CK, and MS designed the study, analyzed the data and interpreted the results. ST, TH, MM, CK, HM, and MS wrote the manuscript. All authors reviewed and discussed the results.

## Funding

TH was partially funded by the Bindley Bioscience Center Fellow program. This research was supported by NIH GM61721 and CIRM RT307678 to CK, the Ruth L. Kirschstein National Research Service Award GM007185 to MM. Research reported in this publication was also supported by the National Institute of Allergy And Infectious Diseases of the National Institutes of Health under Award Number R56AI114861 to MS.

### Conflict of interest statement

The authors declare that the research was conducted in the absence of any commercial or financial relationships that could be construed as a potential conflict of interest.
