# Supplementary material for: Repurposing Approach Identifies Auranofin with Broad Spectrum Antifungal Activity That Targets Mia40-Erv1 Pathway
Source: Front Cell Infect Microbiol. 2017 Jan 18;7:4. doi: 10.3389/fcimb.2017.00004 (PMC5241286; doi:10.3389/fcimb.2017.00004)
Supplement: Supplementary file 2 [file DataSheet2.docx]

|  | Compound (sample) | PCR-product | Primer Name | Primer sequence |
| --- | --- | --- | --- | --- |
| Primer 1 |  | all PCR products | BarseqUp | CAAGCAGAAGACGGCATACGAGCTCTTCCGATCTGCACGTCAAGACTGTCAAGG |
| Primer 2 | DMSO (1.2) | 1 | Miseq_U1_M1 | ATGATACGGCGACCACCGAGATCTACACTCTTTCCCTACACGACGCTCTTCCGATCTaaacaGATGTCCACGAGGTCTCT |
|  | DMSO (1.3) | 2 | Miseq_U1_M2 | ATGATACGGCGACCACCGAGATCTACACTCTTTCCCTACACGACGCTCTTCCGATCTactaaGATGTCCACGAGGTCTCT |
|  | DMSO (1.4) | 3 | Miseq_U1_M3 | ATGATACGGCGACCACCGAGATCTACACTCTTTCCCTACACGACGCTCTTCCGATCTattccGATGTCCACGAGGTCTCT |
|  | Auranofin (4.2) | 10 | Miseq_U1_M10 | ATGATACGGCGACCACCGAGATCTACACTCTTTCCCTACACGACGCTCTTCCGATCTactcgGATGTCCACGAGGTCTCT |
|  | Auranofin (4.3) | 11 | Miseq_U1_M11 | ATGATACGGCGACCACCGAGATCTACACTCTTTCCCTACACGACGCTCTTCCGATCTattgaGATGTCCACGAGGTCTCT |
|  | Auranofin (4.4) | 12 | Miseq_U1_M12 | ATGATACGGCGACCACCGAGATCTACACTCTTTCCCTACACGACGCTCTTCCGATCTcgcacGATGTCCACGAGGTCTCT |

Supplementary Table 1. Primers used for PCR reactions

|  | DMSO | Auranofin (34µg/ml) |
| --- | --- | --- |
| NADH | -0.47 | -0.45 |
| DMSO/Auranofin | -0.52 | -0.64 |
| CCCP | -1.8 | -1.15 |

Supplementary Table 2. Oxygen consumption assay
